# Protein Kinase Cα (PKCα) Regulates Bone Architecture and Osteoblast Activity*

**DOI:** 10.1074/jbc.M114.580365

**Published:** 2014-07-28

**Authors:** Gabriel L. Galea, Lee B. Meakin, Christopher M. Williams, Sarah L. Hulin-Curtis, Lance E. Lanyon, Alastair W. Poole, Joanna S. Price

**Affiliations:** From the ‡ School of Veterinary Sciences, University of Bristol, Bristol BS2 8EJ, United Kingdom and; the § School of Physiology and Pharmacology, University of Bristol, Bristol BS8 1TD, United Kingdom

**Keywords:** Bone, Estrogen, Gaucher Disease, Osteoblast, Protein Kinase C (PKC), Wnt Signaling, Mechanical Loading

## Abstract

Bones' strength is achieved and maintained through adaptation to load bearing. The role of the protein kinase PKCα in this process has not been previously reported. However, we observed a phenotype in the long bones of *Prkca*^−/−^ female but not male mice, in which bone tissue progressively invades the medullary cavity in the mid-diaphysis. This bone deposition progresses with age and is prevented by disuse but unaffected by ovariectomy. Castration of male *Prkca*^−/−^ but not WT mice results in the formation of small amounts of intramedullary bone. Osteoblast differentiation markers and Wnt target gene expression were up-regulated in osteoblast-like cells derived from cortical bone of female *Prkca*^−/−^ mice compared with WT. Additionally, although osteoblastic cells derived from WT proliferate following exposure to estradiol or mechanical strain, those from *Prkca*^−/−^ mice do not. Female *Prkca*^−/−^ mice develop splenomegaly and reduced marrow GBA1 expression reminiscent of Gaucher disease, in which PKC involvement has been suggested previously. From these data, we infer that in female mice, PKCα normally serves to prevent endosteal bone formation stimulated by load bearing. This phenotype appears to be suppressed by testicular hormones in male *Prkca*^−/−^ mice. Within osteoblastic cells, PKCα enhances proliferation and suppresses differentiation, and this regulation involves the Wnt pathway. These findings implicate PKCα as a target gene for therapeutic approaches in low bone mass conditions.

## Teaser

**Background:** Roles of the multifunctional kinase PKCα in bone are unknown.

**Results:** Female *Prkca*^−/−^ mice form bone in their medullary cavities associated with higher osteoblastic differentiation. Bone and spleen changes in *Prkca*^−/−^ mice resemble features of Gaucher disease.

**Conclusion:** PKCα regulates osteoblast differentiation and bone architecture.

**Significance:** PKCα-targeting therapies may benefit low bone mass conditions, including Gaucher disease and osteoporosis.

## Introduction

Age-related failure of bones' intrinsic ability to match their mass and architecture to their functional load bearing results in fragility and increased incidence of fractures characteristic of osteoporosis (1). Part of this process involves thinning of the load-bearing cortices of long bones due to expansion of the medullary cavity in both women and men (2, 3). This weakens the bone structure and predisposes to fracture (4). Deterioration of bone structure is a consequence of chronic failure of the cells that form bone (osteoblasts) to adequately compensate for the activity of those that resorb it (osteoclasts). This may in part be due to aging-related deficiencies in osteoblast proliferation and differentiation (5, 6). The identification of molecular pathways that enhance osteoblast function has led to novel agents entering clinical trials for the treatment of osteoporosis, including, most recently, neutralizing antibodies against the Wnt antagonist sclerostin, which potently inhibits bone formation (7).

Wnt signaling following sclerostin down-regulation naturally occurs in bones subjected to mechanical loading, correlating with subsequent bone formation (8–10). Osteoblast lineage cells' responses to their mechanical loading-engendered strain environment constitute the primary functional determinant of bone mass and architecture (11). The cellular outcomes of mechanical strain-initiated signaling include site-specific activation of bone formation through osteoblast differentiation and proliferation (9, 12, 13). Molecular mechanisms facilitating these responses include ligand-independent functions of the estrogen receptors (ERs)^4^ (14–16) and the Wnt/β-catenin signaling pathway (10, 17–19). Both Wnt and ER signaling are established drug targets for the treatment of osteoporosis. Both of these potently osteogenic pathways are also regulated by the ubiquitous kinase protein kinase Cα (PKCα) (20–23), leading us to hypothesize that PKCα may critically regulate osteoblast function.

PKCα has been implicated in major disease processes (24) in part through its role as a critical regulator of proliferation in various cell types (21, 22, 25), yet its roles in osteoblasts are largely unknown. PKCα regulates ERα activity in osteoblast-like cells (23, 26) and inhibits Wnt/β-catenin signaling in cancer cell types (27, 28). Exposure to mechanical strain rapidly activates PKCα in osteoblast-like cells (29), and PKC signaling has been implicated in the regulation of various mechanically responsive genes, including the osteoblast differentiation marker osteocalcin (30–32). A role for PKCα in regulating osteoblastic cell differentiation has been suggested previously (33). Furthermore, pharmacological activation of PKC signaling rescues defective proliferation of marrow-derived osteoblastic cells in a mouse model in Type I Gaucher disease (34). This disease involves debilitating osteoporosis and architectural deterioration together with hematological abnormalities associated with mutation of the GBA1 gene (34, 35). Generalized inhibition of PKC signaling has been proposed to contribute to the etiology of this disease (34, 36), but the effects of global impairment of PKC isoforms on Gaucher-related phenotypes have not been investigated.

In this study, we characterize the bone phenotype of previously generated *Prkca*^−/−^ mice in which the *Prkca* gene was disrupted by homologous recombination to generate a mouse constitutively lacking expression of PKCα (37). This mouse has been used to demonstrate roles for the gene in a number of tissues. Cardiac contractility has been shown to be increased in *Prkca*^−/−^ mice, and gene deletion protects against heart failure induced by pressure overload and dilated cardiomyopathy (37). PKCα has also been shown to mediate hypertonicity-stimulated urea transport in the collecting ducts of the kidney (38) and has been shown to be critical for secretion of granule contents from platelets (39). Here we show that targeted deletion of *Prkca* leads to a marked increase in endosteal bone formation, progressively invading the medullary cavity at diaphyseal sites of load-bearing bones in female mice. In the experiments reported here, we sought to establish the mechanisms involved by examining the effects on bone architecture *in vivo* of age, gender, loading, and ovariectomy in *Prkca*^−/−^ compared with WT mice and *in vitro* on osteoblast proliferation and differentiation. We also documented similarities in the *Prkca*^−/−^ mice with Gaucher disease in humans.

## EXPERIMENTAL PROCEDURES

### 

#### 

##### Cell Culture and Treatment

17β-estradiol (E2) was from Sigma and dissolved in ethanol. Wnt3a was from Tocris (Bristol, UK) and dissolved in PBS according to the manufacturer's instructions. Calphostin C and phorbol 12-myristate 13-acetate (PMA) were from Tocris and dissolved in ethanol and dimethyl sulfoxide, respectively. Cells were maintained in phenol red-free DMEM containing 10% heat-inactivated FCS (PAA, Somerset, UK), 2 mM l-glutamine, 100 IU/ml penicillin, and 100 IU/ml streptomycin (Invitrogen) (complete medium) in a 37 ºC incubator at 5% CO_2_, 95% humidity as described previously (19).

Cortical long bone-derived mouse osteoblastic cell (CLBOb) extractions from adult female mice were as described previously (6, 15, 19) and were always used at passage 1. For differentiation studies, CLBObs were seeded at 25,000 cells/cm^2^ and treated with or without 50 μm ascorbic acid and 10 mm β-glycerol phosphate. Alkaline phosphatase assays were using *p*-nitrophenyl phosphate Sigma Fast^TM^ according to the manufacturer's instructions and normalized relative to total protein content using the crystal violet method (6, 40, 41). Mineralized nodules fixed in ice-cold methanol on ice for 5 min were air-dried, washed in phosphate-buffered saline, stained in 2% alizarin red solution, pH 4.2, for 5 min, and cleared under running water, also as reported previously (6, 40, 41).

For strain experiments, cells were cultured on custom-made plastic strips, and strain was applied as described previously (6, 30, 42) through a brief period of 600 cycles of four-point bending of the strips with a peak strain of 3,400 μϵ on a Zwick/Roëll materials testing machine (Zwick Testing Machines Ltd., Leominster, UK) with strain rates on and off of ∼24,000 μϵ/s, dwell times on and off of 0.7 s, and a frequency of 0.6 Hz.

To determine the effect of treatment with the PKC activator PMA, cells were cultured for 7 days and then treated twice with 0.1 μm PMA at 12-h intervals and harvested 12 h after the second treatment.

##### Proliferation Studies and Ki-67 Staining

Proliferation studies and Ki-67 staining, including *in situ* cell cycle analysis, were as described previously by our group (6, 19). For proliferation studies, CLBObs were seeded at an initial density of 10,000 cells/cm^2^, whereas Saos-2 cells were seeded at 5,000 cells/cm^2^ and allowed to settle overnight, flooded with complete medium for 24 h, and then serum-deprived in 2% charcoal/dextran-stripped serum overnight before strain or treatment. To determine cell number, random images of DAPI-stained nuclei were taken at ×4 magnification on a Leica DMRB microscope with an Olympus DP72 digital camera, binarized, and automatically analyzed using ImageJ (National Institutes of Health, version 1.46d). Ki-67-positive cells were counted using ImageJ on five randomly chosen images per slide at an original magnification of ×20. Cycle stage analysis was performed on 190 ± 22 positive nuclei/slide imaged at ×40. Key results were independently verified by two observers (G. L. G. and L. B. M.). Representative images of the pattern of Ki-67 distribution in Saos-2 cells and CLBObs in different stages of the cell cycle have been published previously by our group (6, 19).

##### Quantitative Reverse Transcriptase PCR

In order to isolate marrow and bone fractions, bones were rapidly dissected of all surrounding tissues, and the epiphyses were removed. The diaphyses containing marrow were placed upright in custom-made plastic straws inside 2-ml tubes, which were then centrifuged at 10,000 r.p.m. for 10 s at 4 °C. Bones were snap-frozen for RNA extraction with RNEasy Plus Universal kits (Qiagen, Sussex, UK), whereas marrow was lysed directly in RNEasy Plus lysis buffer (Qiagen) and stored at −80 °C.

qRT-PCR was performed as described previously (18, 43). Mouse β_2_-microglobulin (β*2 mg*), osteocalcin, and Wnt-induced secreted protein 2 (*Wisp2*) were as follows: β*2 mg*, ATGGCTCGCTCGGTGACCCT (forward) and TTCTCCGGTGGGTGGCGTGA (reverse); osteocalcin, CTGACCTCACAGATCCCAAGC (forward) and TGGTCTGATAGCTCGTCACAAG (reverse); *Wisp2*, GGTTTCACCTGCCTGCCGCT (forward) and TCACACACCCACTCGGGGCA (reverse).

All other primer sequences were from the Harvard PrimerBank (43) (Table 1). Gene panels representing osteoblast differentiation and Wnt targets were predetermined, and all quantified genes are presented here.

**TABLE 1:** **List of PrimerBank IDs for PCR primers used in this study**

Gene	PrimerBank ID
**Osteoblast markers**	
*Runx2* (also a Wnt target)	225690525b1
Osterix	18485518a1
Collagen 1 A1 (*Col1A1*)	118131144b1
Osteoprotegerin (also a Wnt target)	113930715b1
Receptor activator of NF-κB ligand (*RANKL*)	114842414b1
Osteocalcin	13811695a1

**Wnt targets**	
*cMyc*	293629266b3
Cyclin D1 (*CCND1*)	119672895b1
*Axin2*	158966712b1
*Cyr61*	239937453b1

**Adipocyte markers**	
Peroxisome proliferator-activated receptor γ (*PPAR*γ)	187960104b1
cAMP element-binding protein α (*C/EBP*α)	131886531b2

**Osteoclast markers**	
Receptor activator of NF-κB (*RANK*)	110350008b1
Tartrate-resistant acid phosphatase (*TRAP*)	156151432b1

##### Western Blotting

Cells were cultured as for proliferation studies and lysed in radioimmune precipitation buffer containing protease inhibitors (Sigma). Lysates were sonicated prior to quantification of protein content by a bicinchonic acid assay (Sigma). Lysate protein content was standardized to 500 μg/ml and solubilized in reducing Laemmli sample buffer. Proteins were resolved by SDS-PAGE and then transferred to PVDF membranes. Membranes were blocked with 10% BSA and subjected to immunoblotting with anti-PKCα (New England Biolabs Ltd., Hitchin, UK); anti-PKCβ, -δ, -ϵ, and -θ (BD Biosciences); and anti-PKCγ (Insight Biotechnology Ltd., Wembley, UK) and α-tubulin (Sigma) as a loading control.

##### Histochemistry

Sclerostin immunodetection on decalcified bone sections was as described previously (9). Hematoxylin and eosin (H&E) staining was done following standard protocols.

##### Hematological Analysis

Blood was taken from 4-month-old mice via cardiac puncture into 50 mm EDTA (1:10, v/v). Samples were immediately analyzed on a Horiba Pentra ES60 hematology analyzer (Horiba UK Ltd., Northampton, UK).

##### Determination of Bone Structure and the Effects of Sciatic Neurectomy, Ovariectomy, and Castration

*Prkca*^−/−^ mice were as described previously (39). All procedures complied with the UK Animals (Scientific Procedures) Act 1986 and were reviewed and approved by the University of Bristol ethics committee. Breeding pairs of *Prkca*^+/−^ mice were crossed to generate *Prkca*^−/−^ and *Prkca*^+/+^ for experimentation as littermate matched pairs. Following sacrifice, legs were stored in 70% ethanol, and whole femur or tibia was imaged by microcomputed tomography (μCT) using the SkyScan 1172 system (SkyScan, Kontich, Belgium) with a voxel size of 4.8 μm (110 μm^3^). The scanning, reconstruction, and method of analysis have been reported previously (6, 44) and were performed according to American Society for Bone and Mineral Research guidelines (45).

Sciatic neurectomy, ovariectomy, and castration were performed to investigate the effects of these interventions on intramedullary bone. Sciatic neurectomy was as described previously by our group (44). Female mice were subjected to unilateral sciatic neurectomy of the right tibia at 15 weeks of age and sacrificed 3 weeks later at 18 weeks of age. Ovariectomy was also performed as described previously by our group (46). Female mice were ovariectomized at 8 weeks of age and sacrificed 10 weeks later at 18 weeks of age. Similarly, male mice were castrated at 8 weeks of age and sacrificed 10 weeks later at 18 weeks of age. The left limbs of ovariectomized or castrated mice were compared with the left control limbs of non-operated mice of the same age.

##### Statistical Analysis

Data are presented as means ± S.E. Comparisons between two groups, including comparisons within gender, were by Student's *t* test following Levene's test for homogeneity of variance. Comparisons between more than two groups were by analysis of variance with post hoc Bonferroni or Games-Howel tests. Genotype by age interactions and genotype by intervention interactions were determined by mixed model analysis with Bonferroni adjustment performed in SPSS (version 17).

## RESULTS

### 

#### 

##### PKCα Deletion Causes Age-dependent and Sex- and Site-specific Changes in Bone Structure

**FIGURE 1.**
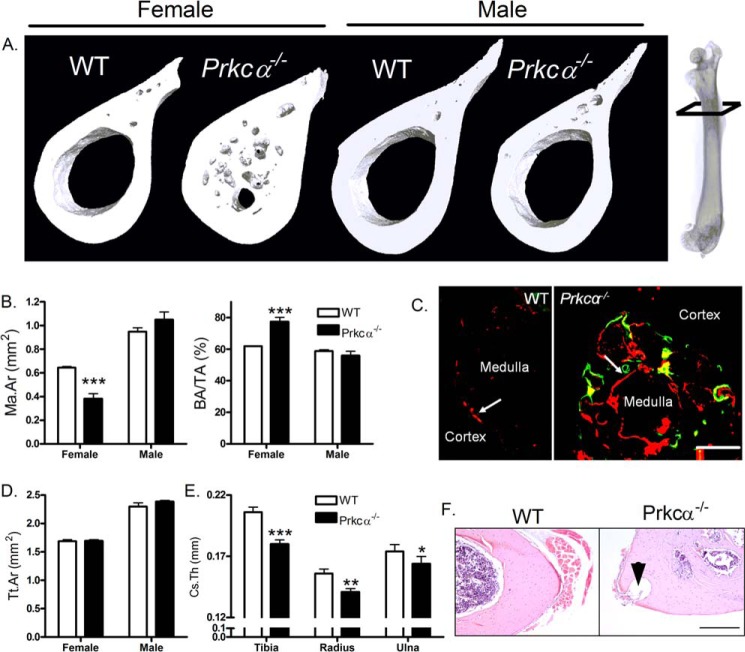
***Prkca* deletion causes intramedullary bone formation in female mice.** *A*, representative three-dimensional μCT reconstructions showing 30% of the bone's length from the proximal end, indicated on the radiograph, in 22-week-old WT and *Prkca*^−/−^ female and male mice. *B*, μCT quantification of medullary area (*Ma.Ar*) and bone area per tissue area (*BA/TA*) in female and male WT and *Prkca*^−/−^ 22-week-old mice (*n* = 5). *C*, dynamic histomorphometry with calcein (*green*) and alizarin (*red*) fluorochromes illustrating medullary double-labeling in the tibia midshaft of 18-week-old female *Prkca*^−/−^ but not WT mice (*scale bar*, 0.5 mm). *D*, total tissue area (*Tt.ar*) was quantified by μCT 30% of the femur's length from its proximal end in 22-week-old male and female WT and *Prkca*^−/−^ mice. *E*, male *Prkca*^−/−^ mice had lower cortical thickness (*Cs.Th*) in the midshaft of the tibia, radius, and ulna than WT males as determined by μCT analysis. *F*, cortical holes, indicated by the *arrowhead*, were observed in all long bones tested in *Prkca*^−/−^ male and female mice. H&E staining illustrates a hole in the humerus of a female *Prkca*^−/−^ (*scale bar*, 1 mm). *, *p* < 0.05; **, *p* < 0.01; ***, *p* < 0.001 *versus* WT of the same gender. *Error bars*, S.E.

Bone structure was investigated by μCT, which revealed that the medullary cavity of female, but not male, *Prkca*^−/−^ mice was characterized by the invasion of disorganized bone in the diaphyses of the femur (Fig. 1*A*), humerus, and tibia. This invasion was sufficient to significantly reduce the area of the medullary cavity and increase bone area fraction (Fig. 1*B*) due to endosteal bone formation (Fig. 1*C*). Total tissue area enclosed within the periosteum was not significantly different between either male or female WT and *Prkca*^−/−^ mice (Fig. 1*D*), suggesting that PKCα specifically influences the endosteal surface. Male *Prkca*^−/−^ mice had reduced cortical thickness in various skeletal sites (Fig. 1*E*), but this parameter could not be accurately assessed in females with extensive intramedullary bone or cortical pores. Both males and females developed large cavities throughout the diaphyseal cortices (Fig. 1*F*). Although reminiscent of the cavities that carry blood vessels in normal bone, these are much larger than any seen in WT mice. No differences in body weight or bone lengths were observed between WT and *Prkca*^−/−^ mice (not shown).

**FIGURE 2.**
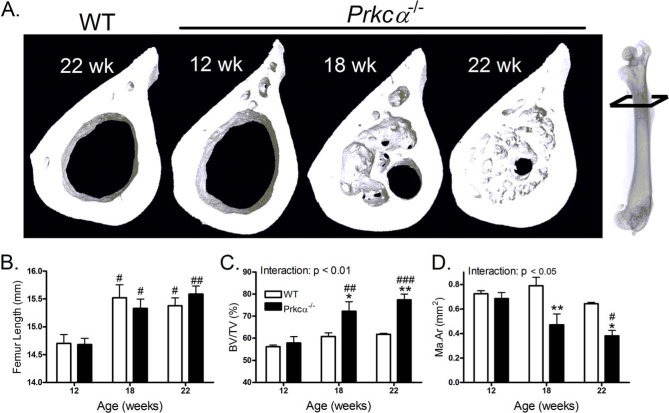
**Intramedullary bone invasion progresses with age in adult female *Prkca*^−/−^ mice.** *A*, representative three-dimensional reconstructions of the femoral 30% site in a 22-week WT mouse and 12-, 18-, or 22-week-old female *Prkca*^−/−^ mice indicating medullary in-filling with age. *B*, quantification of femoral length in female mice of the indicated ages. Shown are quantifications of bone area per tissue area (*BA/TA*) (*C*) and medullary area (*Ma.Ar*) (*D*) in female 12-week-old (*n* = 6), 18-week-old (*n* = 6), and 22-week-old (*n* = 5) mice. Statistical significance of the genotype by age interactions is indicated. #, *p* < 0.05; ##, *p* < 0.01; ###, *p* < 0.001 *versus* 12-week-old mice of the same genotype. *Bars*, mean ± S.E. (*error bars*) *, *p* < 0.05; **, *p* < 0.01 *versus* WT mice of the same age.

The intramedullary bone phenotype was not evident in 12-week-old mice but became evident by 18 weeks (Fig. 2*A*). In both genotypes, 12-week-old female mice had shorter femurs than 18-week-old mice and were thus not skeletally mature (Fig. 2*B*). 12-Week-old *Prkca*^−/−^ mice had significantly lower cortical thickness before intramedullary bone was observed (WT = 0.18 ± 0.006 and *Prkca*^−/−^ = 0.16 ± 0.005, *p* < 0.05). However, the presence of intramedullary bone increased the bone area fraction at 18 weeks and even more so at 22 weeks of age in *Prkca*^−/−^ mice (Fig. 2*C*). This was because of a progressive reduction in medullary area (Fig. 2*D*). Consequently, significant genotype by age interactions were detected by mixed model analysis for both marrow area and bone area fraction (Fig. 2, *C* and *D*).

**FIGURE 3.**
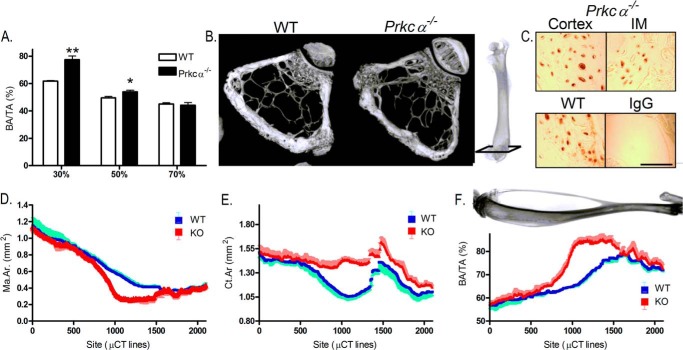
**Intramedullary bone expansion in the bones of female *Prkca*^−/−^ mice occurs at specific sites.** *A*, quantification of bone area per tissue area (*BA/TA*) in three different sites along the length of the femur of 22-week-old female WT and *Prkca*^−/−^ mice (*n* = 5). *B*, representative three-dimensional reconstructions of μCT images of female WT and *Prkca*^−/−^ trabecular bone in the distal femur. Images represent 100 μCT lines taken 100 lines above the growth plate, approximately indicated on the radiograph. *C*, immunolocalization of sclerostin in the humeral diaphysis from a female *Prkca*^−/−^ mouse, indicating sclerostin expression in both the IM and cortical bone. WT cortical bone is shown as a positive control, and a non-immune IgG is shown as a negative control. *Scale bar*, 250 μm. *D–F*, approximately 2,000 measurements were made along the length of the tibia of female WT or *Prkca*^−/−^ mice (*n* = 3 in each case), with each measurement representing a single μCT line (4.8 μm). Shown are medullary area (*Ma.Ar*) (*D*), cortical area (*Ct.Ar*) (*E*), and bone area per tissue area (*BA/TA*) (*F*). *Bars* and *dots*, mean ± S.E. (*error bars*). *, *p* < 0.05; **, *p* < 0.01; ***, *p* < 0.001 *versus* WT at the same site.

Intramedullary bone was consistently localized to ∼30% of the femur's length from the proximal end and did not extend into the cancellous regions at the bones' ends (Fig. 3, *A* and *B*). Expression of the mature osteocyte product sclerostin was detected by immunohistochemistry, indicating the presence of mature osteocytes within the intramedullary bone (Fig. 3*C*). A similar distribution was observed in the tibia, where we fully characterized the location of this intramedullary bone by adapting the method of μCT analysis such that 2,000 cross-sectional measurements were made along the length of the tibia between 20 and 80% of the bone's length. This demonstrated a significant deviation in bone area due to bony invasion of the tibial medullary cavity only in the mid-diaphysis (Fig. 3, *D–F*). The distribution of intramedullary bone in the tibia approximately corresponds to the region of bone predicted by ourselves and others to experience the greatest bending (strain) during axial load bearing (10, 47).

##### Prkca Deletion Parallels Aspects of Gaucher Disease

**FIGURE 4.**
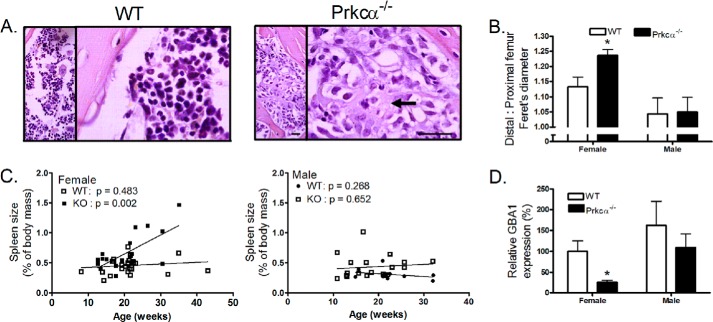
**Deletion of *Prkca*^−/−^ mimics features of type 1 Gaucher disease in female mice.** *A*, representative images of female WT and *Prkca*^−/−^ bone stained with hematoxylin and eosin showing Gaucher-like cells in the *Prkca*^−/−^ marrow in the same region as the IM bone infiltration. *Scale bar*, 50 μm. *B*, Feret's diameter was calculated 25% (proximal) and 75% (distal) of the femur's length from its proximal end. The ratio of distal to proximal in 22-week-old mice of each genotype is shown. *C*, spleen weights of WT and *Prkca*^−/−^ mice of each sex, sacrificed at different ages for different uses, expressed as a proportion of body weight. *p* values shown are for the slope indicating progression with age only in the female *Prkca*^−/−^. *D*, marrow from 22-week-old WT and *Prkca*^−/−^ mice was harvested, and *Gba1* expression was analyzed by qRT-PCR. *Bars*, mean ± S.E. (*error bars*) (*n* = 5). *, *p* < 0.05 *versus* WT.

Sites of intramedullary bone and surrounding marrow were further characterized by histology. Within the marrow, amorphous eosinophilic cells reminiscent of Gaucher cells (48) were observed in regions with intramedullary bone (Fig. 4*A*). Type I Gaucher disease is a condition associated with debilitating skeletal pathologies (49) and has previously been suggested to involve suppression of PKC signaling (34, 36). Other similarities with Gaucher disease observed in *Prkca*^−/−^ mice, including reduction in cortical thickness described above and impaired platelet aggregation previously reported (39), led us to investigate further parallels. Platelet number has previously been reported not to be significantly different between WT and *Prkca*^−/−^ mice (50), and further hematological analysis revealed no significant differences in circulating total white blood cell numbers between WT (*n* = 4) and *Prkca*^−/−^ (*n* = 8) mice (*Prkca*^−/−^ 96 ± 5% of WT, *p* = 0.28).

Human Gaucher patients develop a distinct “Erlenmeyer flask” deformity in which the proximal femur narrows relative to the distal femur (49). The ratio of Feret's diameter (maximum diameter) between the distal and proximal femur was greater in female *Prkca*^−/−^ than in WT mice, indicating a similar narrowing of the proximal relative to the distal femur (Fig. 4*B*). Splenomegaly is another common feature of Gaucher disease (49). Progressive, age-related splenomegaly was observed in female but not male *Prkca*^−/−^ mice (Fig. 4*C*). To determine whether *Prkca* deletion alters expression of the gluocerebrosidase 1 (*Gba1*) gene causally mutated in Gaucher disease (49), bone marrow was collected from 22-week-old male and female *Prkca*^−/−^ mice, and *Gba1* expression was quantified by qRT-PCR. *Gba1* expression was lower in marrow from female but not male *Prkca*^−/−^ mice relative to WT (Fig. 4*D*).

##### PKCα Influences the Balance between Osteoblastic Proliferation and Differentiation in Vitro

Marrow-derived osteoblastic cells from a mouse model of Gaucher disease have previously been reported to have impaired proliferation that could be rescued by PKC activation (34). In order to investigate whether differences in osteoblast proliferation and differentiation might contribute to the bony invasion of the medullary cavity in female Prkca^−/−^ mice, we investigated the effect of *Prkca* deletion on primary cultures of osteoblast-like cells derived from the load-bearing cortices of mouse long bones (CLBObs). CLBObs have been extensively characterized by our group and are able to respond to osteogenic stimuli, including Wnts, estradiol, and physiological mechanical strain (15, 18, 19, 31, 47, 51, 52). Robust PKCα expression was detected in CLBObs from WT mice, whereas no expression was detected in cells from *Prkca*^−/−^ mice by Western blotting (data not shown). None of the other PKC isoforms tested (PKCβ, PKCγ, PKCσ, and PKCϵ) were expressed differently between cells of the two genotypes (data not shown). PKCθ was not detected in either genotype (data not shown).

**FIGURE 5.**
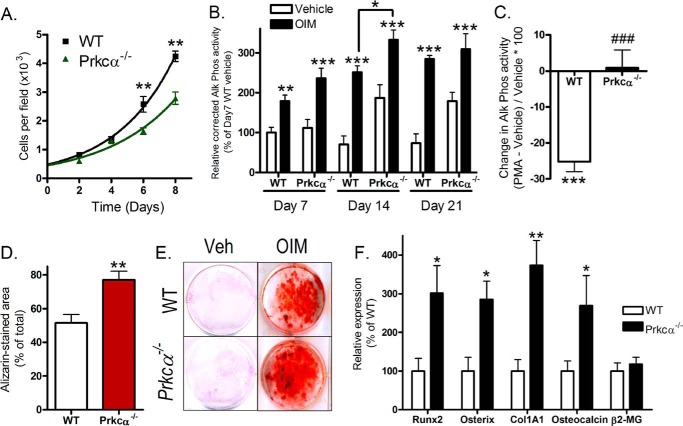
**Osteoblast-like cells from female *Prkca*^−/−^ mice have an enhanced differentiation state *in vitro*.** CLBObs were derived from female WT and *Prkca*^−/−^ mice. *A*, growth curves were determined by counting the cell number at the indicated time points. *B*, CLBObs derived from WT and *Prkca*^−/−^ mice were cultured for the indicated period of time with or without treatment with osteogenesis induction medium (*OIM*). Alkaline phosphatase activity was determined and normalized to total protein content (*n* = 12). Unless indicated, comparisons are relative to vehicle-treated cultures of the same genotype at the same time point. *C*, cultures from WT and *Prkca*^−/−^ mice were treated with the PKC activator PMA. Alkaline phosphatase activity was determined normalized to total protein content, and the percentage change in activity relative to vehicle-treated controls is shown. *D*, quantification of the proportion of culture area stained with alizarin red after 21 days of treatment with osteogenesis induction medium (*n* = 12). *E*, representative cultures from WT and *Prkca*^−/−^ mice fixed following 21 days of treatment with vehicle (*veh*) or OIM and stained with alizarin red. *F*, qRT-PCR quantification of osteoblastic differentiation markers in CLBObs after 14 days of culture (*n* = 12). β2-MG housekeeping gene expression is shown per μg of RNA. *Bars*, mean ± S.E. (*error bars*). *, *p* < 0.05; **, *p* < 0.01; ***, *p* < 0.001 *versus* WT controls. ###, *p* < 0.001 *versus* the percentage change in WT cultures.

CLBObs from adult female *Prkca*^−/−^ mice were less proliferative than those in similar cultures derived from WT mice (Fig. 5*A*). Ki-67 *in situ* cell cycle analysis revealed that this was due to a greater proportion of cells from *Prkca*^−/−^ mice being in a Ki-67-negative, quiescent state with no significant differences observed between proliferating cells in different stages of the cell cycle (not shown). Consistent with a shift in the proliferation-differentiation balance away from proliferation, CLBObs from female *Prkca*^−/−^ mice showed spontaneously increased activity of the early osteoblastic differentiation marker alkaline phosphatase by 14 days of culture (Fig. 5*B*). Osteogenesis induction medium increased alkaline phosphatase activity further in both WT and *Prkca*^−/−^ cultures (Fig. 5*B*), whereas activation of PKC signaling by phorbol ester PMA (0.1 μm) reduced alkaline phosphatase activity in WT but not *Prkca*^−/−^ cultures (Fig. 5*C*). By day 21 of treatment with osteogenesis induction medium, cells from female *Prkca*^−/−^ mice had mineralized a greater proportion of their cell culture surface than cells from WT mice (Fig. 5, *D* and *E*). Expression of markers of osteoblastic differentiation was also higher in CLBOb cultures from *Prkca*^−/−^ than in those from WT mice (Fig. 5*F*).

**FIGURE 6.**
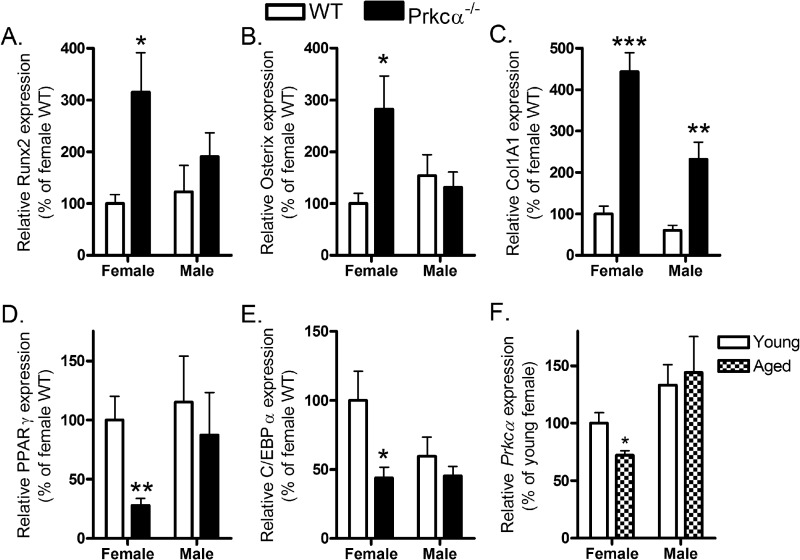
**Prkca deletion alters marrow expression of differentiation markers in a sex-specific manner.** Marrow was harvested from pooled tibiae and femurs of male and female WT and *Prkca*^−/−^ mice and processed by qRT-PCR analysis of the osteoblastic differentiation markers *Runx2* (*A*), osterix (*B*), and *Col1A1* (*C*) and the adipogenic markers *PPAR*γ (*D*) and *C/EBP*α (*E*) (*n* = 5). *F*, *Prkca* expression was quantified in marrow from young adult (17-week-old) or aged (19-month-old) male and female mice (*n* = 8). *Bars*, mean ± S.E. (*error bars*). *, *p* < 0.05; **, *p* < 0.01; ***, *p* < 0.001 *versus* the respective WT controls.

To validate these findings *in vivo*, osteoblastic differentiation markers were quantified in both marrow and cortical bone extracted from 22-week-old female and male *Prkca*^−/−^ mice. *Runx2* and osterix expression was significantly elevated in marrow from female, but not male, *Prkca*^−/−^ mice (Fig. 6, *A* and *B*), whereas collagen 1 A1 (*Col1A1*) was elevated in *Prkca*^−/−^ mice of both genders (Fig. 6*C*). Surprisingly, none of these differences were observed in the bone tissue predominantly representing terminally differentiated osteocytes; nor were osteoclast-related markers differently expressed (not shown). Consistent with enhanced osteoblast lineage commitment at an early stage of differentiation, markers of adipogenic differentiation were significantly lower in marrow from female but not male *Prkca*^−/−^ mice (Fig. 6, *D* and *E*). We also detected an effect of age on *Prkca* expression; levels were lower in marrow of 19-month-old (aged) female, not male, WT mice compared with levels in 18-week-old (adult) mice (Fig. 6*F*).

**FIGURE 7.**
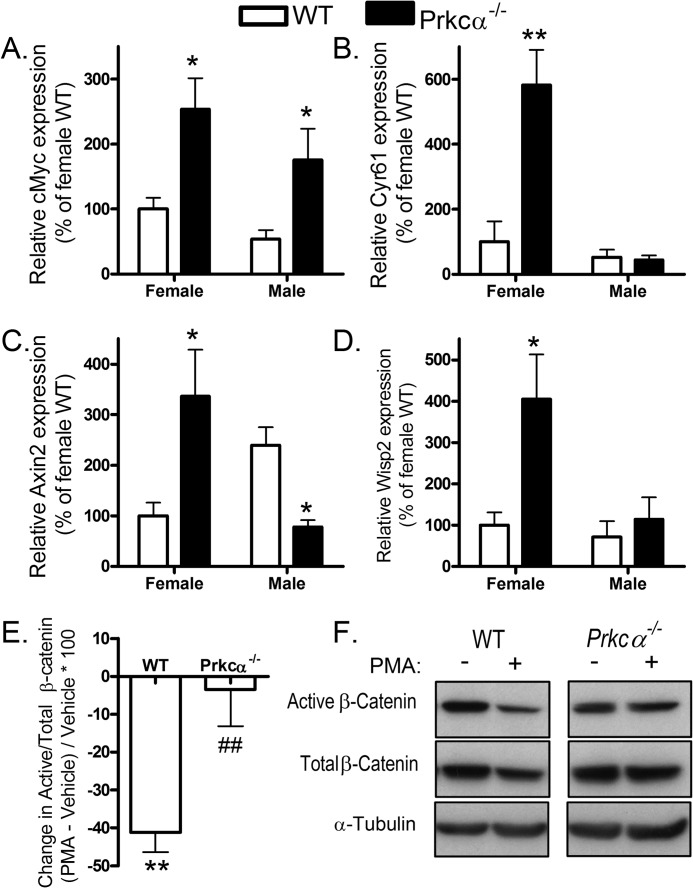
**Prkca suppresses Wnt/β-catenin signaling.** *A–D*, marrow was harvested from pooled tibiae and femurs of male and female WT and Prkca^−/−^ mice and processed by qRT-PCR analysis of the proliferation marker *cMyc* (*A*) and other direct Wnt target genes *Cyr61* (*B*), *Axin2* (*C*), and *Wisp2* (*D*) (*n* = 5). *E* and *F*, CLBObs from WT and *Prkca*^−/−^ female mice were treated with a 0.1 μm concentration of the PKC activator PMA twice at 12-h intervals and lysed 12 h following the second treatment. Active (dephosphorylated) and total β-catenin levels were determined by Western blotting relative to α-tubulin. *E*, the percentage change in active *versus* total β-catenin was calculated (*n* = 6). *F*, representative Western blots. *Bars*, mean ± S.E. (*error bars*). *, *p* < 0.05; **, *p* < 0.01 for the effect of PMA treatment; ##, *p* < 0.01 *versus* the change in WT controls.

Because the Wnt pathway is a critical regulator of osteoblast differentiation, we investigated whether Wnt signaling was altered in *Prkca*^−/−^ mice by quantifying the expression of selected Wnt target genes in marrow and bone from *Prkca*^−/−^ and WT mice. The proliferating cell marker *cMyc* was overexpressed in marrow from both female and male *Prkca*^−/−^ mice (Fig. 7*A*). *Cyr61*, which is involved in the promotion of osteoblast differentiation by Wnts (53), was dramatically elevated in female but not male *Prkca*^−/−^ marrow (Fig. 7*B*). Female *Prkca*^−/−^ marrow had elevated, whereas males had reduced, expression of *Axin2* (Fig. 7*C*), and female, but not male, *Prkca*^−/−^ marrow had elevated expression of *Wisp2* relative to WT (Fig. 7*D*). Thus, in the marrow of female mice, deletion of PKCα up-regulated the expression of all Wnt target genes tested. Furthermore, PKC activation with PMA reduced the proportion of β-catenin in the active (dephosphorylated) form in CLBObs from WT but not *Prkca*^−/−^ mice (Fig. 7, *E* and *F*), demonstrating that PKCα suppresses active β-catenin in a manner that cannot be redundantly achieved by activation of other PKC isoforms in these cells.

##### PKCα Promotes Osteoblastic Proliferation in Vitro following Strain and Estradiol, not Wnt3a

**FIGURE 8.**
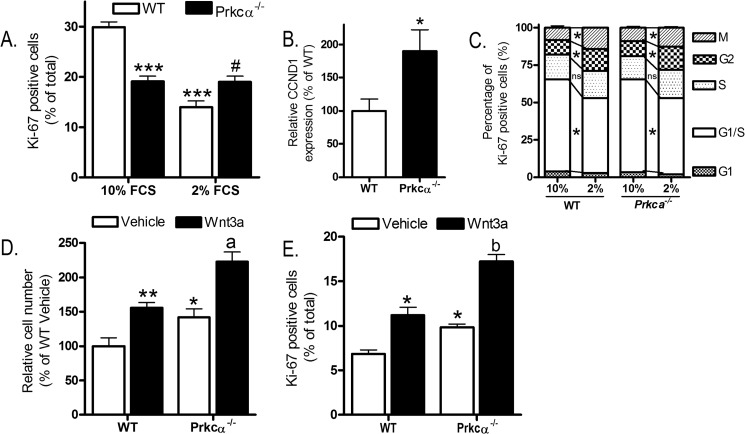
***Prkca* deletion alters recruitment of osteoblast-like cells to the cell cycle but not Wnt3a-induced proliferation.** *A*, CLBObs female from WT and *Prkca*^−/−^ mice were cultured under permissive (10% FCS) or serum-depleted (2% c/d FCS, used for subsequent proliferation studies) for 24 h. The proportion of cells stained positive for Ki-67 was determined. *B*, CCND1 expression was quantified in qRT-PCR in serum-depleted (2% c/d FCS) subconfluent cultures of CLBObs from WT and *Prkca*^−/−^ female mice (*n* = 6). *C*, the proportion of Ki-67-positive cells with a pattern of staining consistent with the indicated cell cycle stages was determined in CLBObs from female WT and *Prkca*^−/−^ mice cultured under permissive (10% FCS) or serum-depleted (2% c/d FCS, used for subsequent proliferation studies) for 24 h (*n* = 8). Cells were treated with Wnt3a and fixed 48 h later for cell number analysis (*C*) or 24 h later for Ki-67 analysis (*D*). *Bars*, mean ± S.E. (*error bars*). *, *p* < 0.05; **, *p* < 0.01; ***, *p* < 0.001 *versus* WT controls. #, *p* < 0.05 *versus* WT with 2% FCS. *a*, *p* < 0.05; *b*, *p* < 0.01 *versus* Wnt3a-treated cells from *Prkca*^−/−^ mice.

To investigate the role of PKCα in proliferation of osteoblastic cells following mitogenic stimuli, CLBObs were first serum-depleted in 2% charcoal/dextran-stripped (c/d) fetal calf serum. As expected (54), this reduced proliferation of WT CLBObs but surprisingly had no effect on the proportion of cells stained positive for Ki-67 in cultures from female *Prkca*^−/−^ mice (Fig. 8*A*). Expression of the proliferating cell marker cyclin D1 was confirmed to be greater in serum-depleted CLBObs from *Prkca*^−/−^ than in those from WT mice (Fig. 8*B*). Serum depletion altered the distribution of proliferating cells in the cell cycle similarly in both genotypes, such that the only difference between the genotypes was the initial step whereby quiescent cells become Ki-67-positive (Fig. 8*C*). Treatment with Wnt3a (10 ng/ml), known to increase osteoblastic cell proliferation (19), similarly increased cell number and the proportion of cells stained for Ki-67 in both genotypes (Fig. 8, *D* and *E*).

**FIGURE 9.**
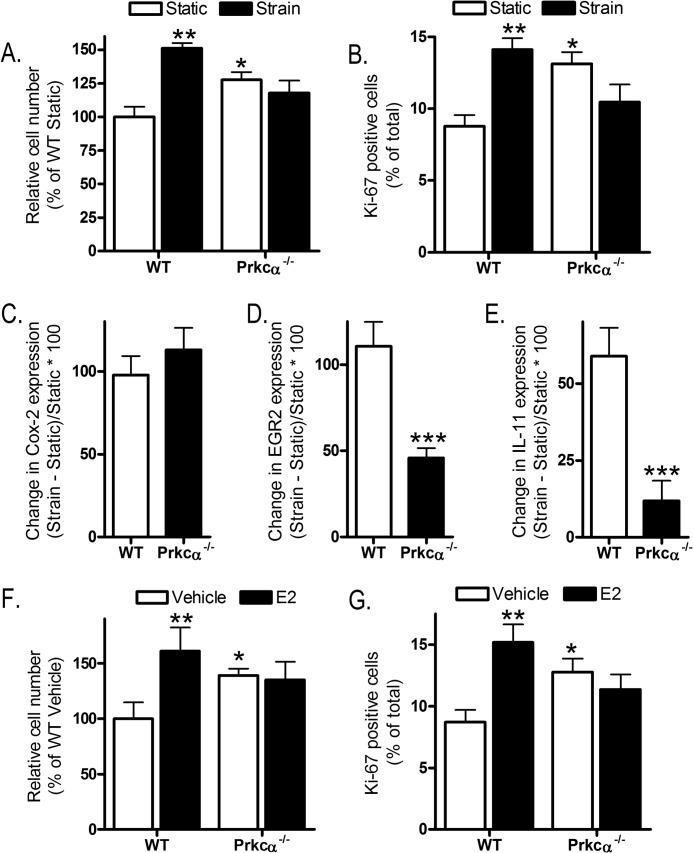
***Prkca* deletion prevents increased proliferation following mechanical strain or estradiol and alters strain-related gene regulation.** CLBObs from female WT and *Prkca*^−/−^ mice were subjected to strain and fixed 48 h later for cell number analysis (*A*) or 24 h later for Ki-67 analysis (*B*). *C* and *E*, cells were subjected to strain or kept as static controls and harvested 1 h later to quantify *Cox-2* (*C*), *EGR2* (*D*), and *IL-11* (*E*). The percentage changes in expression in strained *versus* static control slides are shown (*n* = 12–15). *F* and *G*, cells were treated with 0.1 μm E2 and fixed 48 h later to count cell number or 24 h later for Ki-67 analysis. *Bars* for proliferation data represent the mean ± S.E. (*error bars*) (*n* = 8). *, *p* < 0.05; **, *p* < 0.01; ***, *p* < 0.001 *versus* WT controls.

Given that canonical Wnt signaling is activated in osteoblastic cells subjected to mechanical strain (18) and contributes to the mechanisms by which strain induces osteoblastic cell proliferation (19, 53, 55), we exposed cells to mechanical strain by four-point bending of their substrate. This increased proliferation of WT-derived CLBObs, as expected (19, 53, 55), but did not increase proliferation of cells similarly derived from *Prkca*^−/−^ mice (Fig. 9, *A* and *B*). The involvement of PKCα in strain-responsive signaling was further investigated by comparing the expression of the known strain target genes *Cox-2*, *Egr2*, and *IL-11* (15, 31). In both genotypes, *Cox-2* was up-regulated to a similar degree 1 h after strain (Fig. 9*C*). In contrast, although *Egr2* was significantly up-regulated in both genotypes, this was to a significantly lower extent in cells from *Prkc*a^−/−^ mice (Fig. 9*D*). *IL-11* was up-regulated in CLBObs from WT but not *Prkc*a^−/−^ mice (Fig. 9*E*).

Intriguingly, this pattern of gene regulation following strain is similar to that observed in CLBObs lacking the activation function 1 domain of ERα (15), the receptor that facilitates osteoblast proliferation following both strain and estradiol treatment (19, 56). Estradiol treatment (0.1 μm) was unable to increase cell number or Ki-67 positivity in CLBObs from *Prkca*^−/−^ mice as it did in cultures from WT mice (Fig. 9, *F* and *G*). Treatment with Wnt3a, strain, or estradiol did not alter the cell cycle distribution of proliferating cells in either genotype (not shown).

**FIGURE 10.**
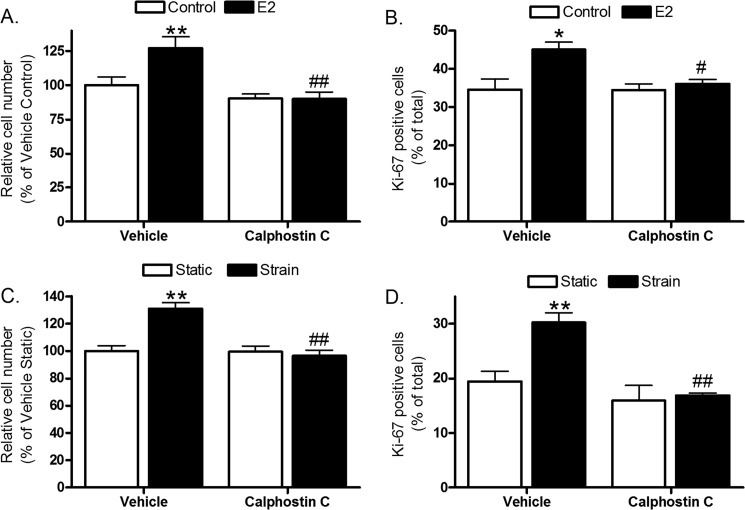
**PKC inhibition prevents human osteoblastic cell proliferation following strain and estradiol.** Saos-2 cells were treated with 1 μm E2 (*A* and *B*) or subjected to strain with or without 30-min pretreatment with 0.1 μm photoactivated calphostin C (*C* and *D*) and fixed 36 h later for cell number analysis (*A* and *C*) or 24 h later for Ki-67 analysis (*B* and *D*). *Bars*, means ± S.E. (*error bars*) (*n* = 8). *, *p* < 0.05; **, *p* < 0.01 *versus* vehicle controls. #, *p* < 0.05; ##, *p* < 0.01 *versus* the *second bar* in each *graph*.

PKC involvement in estradiol- and strain-induced osteoblast proliferation was substantiated in cells of the human female osteoblastic Saos-2 line in which PKC signaling was blocked by pretreatment with 0.1 μm photoactivated calphostin C before strain or estradiol treatment (Fig. 10, *A–D*). Thus, osteoblast proliferation in response to these anabolic stimuli is impaired when PKC signaling is inhibited.

##### Disuse Prevents Intramedullary Bone Invasion in Female Prkca^−/−^ Mice

**FIGURE 11.**
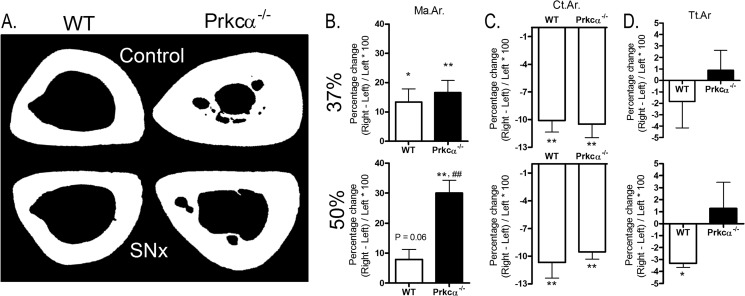
**Disuse influences intramedullary bone in the tibia of female *Prkca*^−/−^ mice.** Female *Prkca*^−/−^ mice and WT controls (*n* = 6) were subjected to unilateral right sciatic neurectomy (*SNx*), causing disuse of the right tibia, at 15 weeks of age and sacrificed 3 weeks later. Left limbs served as internal controls. The effect of sciatic neurectomy was determined at 37 and 50% of the bone's length from the proximal end. *A*, representative cross-sectional images are shown at the 50% site. The percentage change in medullary area (*Ma.Ar*) (*B*), cortical area (*Ct.Ar*) (*C*), and total tissue area (*Tt.Ar.*) (*D*) was determined by comparison with the left control limbs by μCT. *Bars*, mean percentage change ± S.E. (*error bars*). *, *p* < 0.05; **, *p* < 0.01, indicating the effect of neurectomy. ##, *p* < 0.01; ###, *p* < 0.001 *versus* the percentage change in WT mice.

The inability of osteoblast-like cells from *Prkca*^−/−^ mice to proliferate following strain or estradiol treatment, together with the gender and site specificity of the IM bone phenotype in *Prkca*^−/−^ mice, led us to hypothesize that IM bone formation may be related to load bearing and/or circulating estrogens. To investigate the influence of load bearing, we substantially reduced it from the right tibiae of *Prkca*^−/−^ and WT mice by unilateral sciatic neurectomy and compared subsequent bone mass with that in the contralateral left tibia, which acted as an internal control. The effect of *Prkca* deletion on the response to disuse was assessed at 37% of the bone's length from the proximal end, as reported previously (44). At this site, the bone structure was similar between WT and *Prkca*^−/−^ mice, and the absence of PKCα in *Prkca*^−/−^ mice did not influence bone loss (Fig. 11, *A–D*), which was not significantly different from that in WT at this site.

However, at the 50% site where IM bone is present in the *Prkca*^−/−^ mice, disuse resulted in significantly less IM bone (Fig. 11*A*), such that the medullary area of neurectomized limbs in *Prkca*^−/−^ mice was not significantly different from that of the control limbs of WT mice (*p* = 0.16). The overall reduction in bone area was similar between the two genotypes due to significantly smaller total tissue area in the disused limb of WT but not *Prkca*^−/−^ mice (Fig. 11, *B* and C). This suggests that the invasion of the medullary cavity by intramedullary bone in female *Prkca*^−/−^ mice is promoted by load bearing.

**FIGURE 12.**
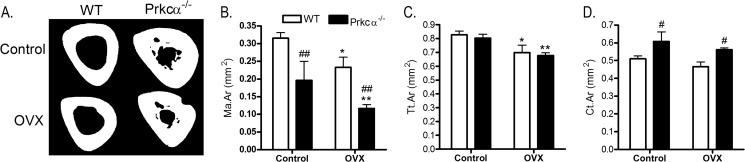
**Ovariectomy does not prevent intramedullary bone in the tibia of female *Prkca*^−/−^ mice.** Female *Prkca*^−/−^ mice (*n* = 7) and WT controls (*n* = 5) were subjected to ovariectomy (*OVX*) at 8 weeks of age and sacrificed 10 weeks later. Their left limbs were compared with the left limbs of non-ovariectomized controls. *A*, representative cross-sectional images are shown. Medullary area (*Ma.Ar*; *B*), total tissue area (*Tt.Ar*; *C*), and cortical area (*Ct.Ar*; *D*) were determined by μCT 50% of the tibia's length from the proximal end. *Bars*, mean percentage change ± S.E. (*error bars*). *, *p* < 0.05; **, *p* < 0.01, indicating the effect of OVX. #, *p* < 0.05; ##, *p* < 0.01 *versus* similarly treated WT mice.

The effect of ovarian hormones on IM bone formation was investigated by subjecting young female mice to ovariectomy (OVX). OVX was performed at 8 weeks of age, before IM bone forms, and the presence of IM bone was analyzed 10 weeks later. OVX resulted in smaller marrow area and smaller total tissue area in both WT and *Prkca*^−/−^ mice (Fig. 12, *A–D*). These effects of OVX were similar in both genotypes (genotype by OVX interaction, *p* > 0.8, as determined by mixed models). Bone area in OVX *Prkca*^−/−^ mice was greater than in non-ovariectomized wild-type mice (*p* < 0.05), demonstrating that loss of ovarian hormones did not prevent IM bone development (Fig. 12*A*).

**FIGURE 13.**
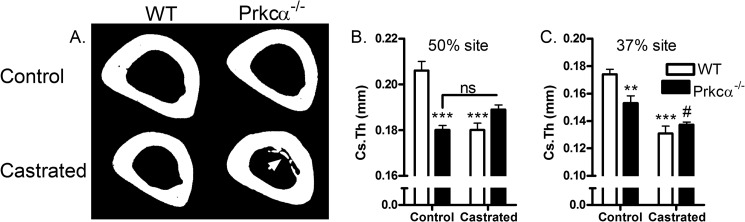
**The cortical response to castration is site-specifically altered in *Prkca*^−/−^ mice due to intramedullary bone formation in the tibial midshaft.** *A*, representative two-dimensional μCT images of control and castrated male WT and *Prkca*^−/−^ mice. The *arrow* indicates the presence of intramedullary bone only observed in *Prkca*^−/−^ mice. Cortical thickness (*Cs.Th*) was quantified in the tibial midshaft (50%) (*B*) and the proximal 37% site (*C*). *Bars*, mean ± S.E. (*error bars*); controls, *n* = 6; WT castrated, *n* = 4; *Prkca*^−/−^ castrated, *n* = 6. *ns*, not significant. **, *p* < 0.01, *** *p* < 0.001 *versus* WT control. #, *p* < 0.05 *versus* control *Prkca*^−/−^ mice.

We therefore next investigated whether the lack of intramedullary bone in the long bones of male *Prkca*^−/−^ mice is due to androgens in males rather than high levels of ovarian steroids in females. 10 weeks following castration, small amounts of intramedullary bone were observed in the tibia of *Prkca*^−/−^ but none in WT mice (Fig. 13*A*). Remarkably, this IM bone in male mice occurred in the tibial midshaft (50% of the bone's length) at the same site as it did in female *Prkca*^−/−^ mice. Castration significantly reduced tibial midshaft cortical thickness in the WT but not *Prkca*^−/−^ mice, potentially due to the presence of intramedullary bone at this site (Fig. 13*B*). At the proximal 37% site of the tibia without intramedullary bone, castration reduced cortical thickness in both WT and *Prkca*^−/−^ mice (Fig. 13*C*). The presence of IM bone was not sufficient to significantly alter marrow area or cortical bone area relative to castrated WT mice (not shown).

## DISCUSSION

Our observation of a bone phenotype in female mice with *Prkca* deletion led us to investigate the potential involvement of PKCα in key osteoregulatory signaling pathways in whole bones *in vivo* and in osteoblasts *in vitro. Prkca* deletion had no effect on the medullary cavity in young mice, but this situation changed with maturity between 12 and 22 weeks of age when female, but not male, *Prkca*^−/−^ mice formed diaphyseal intramedullary bone in various long bones, leaving their periosteal dimensions unaffected. This phenotype is thus remarkable for its age and gender specificity as well as its consistent presence in restricted bone sites.

Histological investigation of these sites led us to identify Gaucher-like cells in the bone marrow in affected regions of the medullary cavity. Various other recognized features of Gaucher disease have been documented, predominantly in female *Prkca*^−/−^ mice, including marrow infiltration, loss of GBA1 expression, splenomegaly, reduced cortical thickness, bone vascular changes, and impaired platelet aggregation (35, 39, 57, 58). Inhibition of PKC signaling due to sphingolipid accumulation is believed to contribute to the etiology of Gaucher disease (36), and our findings are consistent with this hypothesis, demonstrating that selective global deletion of the *Prkca* isoform is sufficient to mimic aspects of the disease. However, loss of PKCα cannot explain all features of Gaucher disease, including the increase in bone resorption in patients (35) compared with the predominant phenotype of deregulated endosteal formation in these mice. Thus, although we do not consider *Prkca*^−/−^ mice a model of Gaucher disease, they may provide insights into its pathogenesis, particularly with relation to bone involvement previously suggested to involve PKC (34).

Osteoblastic cells from a mouse model of Gaucher disease had previously been reported to have deficits in their differentiation and proliferation (34). In the present study, osteoblast differentiation markers were higher in the marrow of *Prkca*^−/−^ mice relative to their WT counterparts, and increased osteoblastic differentiation was also observed *in vitro* using osteoblastic cells derived from the long bones of *Prkca*^−/−^ mice relative to WT. Consistent with a switch in the balance from osteoblast proliferation toward differentiation, osteoblastic cells from *Prkca*^−/−^ mice were less proliferative than WT-derived cells under permissive culture conditions. The initial step of recruitment to the cell cycle was the only difference observed between the two genotypes, illustrating the role of PKCα as a signaling node promoting proliferation in response to numerous mitogenic stimuli. However, osteoblastic cells from *Prkca*^−/−^ mice retain the ability to increase their proliferation, as demonstrated by their response to Wnt3a, which was similar to that observed in cells derived from WT mice.

Exposure to a short period of dynamic mechanical strain change did not increase proliferation of cells from *Prkca*^−/−^ mice as it did in WT-derived cells, and the strain-related up-regulation of EGR2 and IL-11 was also deficient in osteoblastic cells lacking *Prkca*. Given that it is the responses of such resident bone cells to the strains experienced during habitual loading that determine bone architecture (11), perturbation of the signaling axes involved may account for the reduction in cortical thickness and Erlenmeyer flask-like architecture observed in *Prkca*^−/−^ mice, which parallel changes in Gaucher patient femora (35).

However, not all responses to strain are dependent on PKCα because *Cox-2* up-regulation by strain was similar in both genotypes. This pattern of *Cox-2*, *Egr2*, and *IL-11* regulation following strain observed in cells from Prkca^−/−^ mice is similar to the regulation of these genes in osteoblastic cells lacking the activation function 1 domain of ERα (15). Given that the ERα activation function 1 domain mediates its interactions with other proteins, including PKC (59), these findings suggest that PKCα and ERα contribute to the same signaling pathways initiated in osteoblastic cells by strain. ERα is also required for osteoblastic cells to increase their proliferation following strain and following estradiol treatment (19, 56), and deletion of *Prkca* also prevented osteoblastic proliferation following strain or estradiol treatment in the present study. PKCα and ERα physically interact in osteoblastic cells in a complex involving c-Src (23), suggesting a potential mechanism for cooperation between these proteins within the same signaling cascades. Furthermore, PKC signaling reduces ER signaling in overconfluent osteoblastic cells, which attain a more differentiated state (26, 60), potentially acting as a cellular context-specific “break” on ER signaling that is lost in *Prkca*^−/−^ mice. Although none of the myriad ERα transgenic mice generated thus far have been reported to form intramedullary bone, it is intriguing that ERα deletion only impairs the osteogenic response to loading in female, not male, mice (15, 61). This sex-specific facilitation of bone's adaptation to loading by ERα is ligand-independent and non-genomic (15), suggesting that it involves interactions with signaling molecules potentially including PKCα.

Because it is well established that sex hormones and mechanical loading both involve signaling through the ERs (15, 53, 61), we investigated the effect of load bearing on bone structure in *Prkca*^−/−^ mice through sciatic neurectomy-induced disuse and the potential role of systemic ovarian hormones through castration or ovariectomy prior to the development of intramedullary bone. The change in cortical bone area triggered by disuse was not influenced by loss of PKCα, suggesting that the increase in resorption due to disuse is not significantly impaired by loss of PKCα. However, disuse prevented any significant invasion of the medullary cavity in female *Prkca*^−/−^ mice. Based on our current studies, we cannot exclude the possibility that, in addition to inducing disuse, sciatic neurectomy prevented medullary invasion by reducing sympathetic stimulation. Although some authors have reported that the sympathetic nervous system is involved in bone loss caused by hind limb suspension (62, 63), sympathetic blockade does not alter bone gain following loading or bone loss due to neurectomy-induced disuse (64). Disuse, be it through sciatic neurectomy or hind limb suspension, reduces Wnt signaling (9, 65). Thus, an alternative hypothesis is that disuse prevents medullary bone formation by locally suppressing the increase in Wnt signaling observed in the bones of female *Prkca*^−/−^ mice relative to WT.

In *Prkca*^−/−^ males, castration resulted in intramedullary bone formation at the same skeletal sites where it was observed in female *Prkca*^−/−^ mice. However, the amount of intramedullary bone formed in castrated male *Prkca*^−/−^ mice was considerably less than in female *Prkca*^−/−^ mice of the same age. This is not surprising because bone had only 10 weeks to form (from castration to sacrifice). Androgen signaling has previously been reported to suppress bone's response to loading (66), which could explain this result and supports our conclusion that PKCα signaling may influence bone's response to load bearing. This situation contrasts with that following ovariectomy, which did not alter intramedullary invasion. Thus, site-specific intramedullary bone formation in female *Prkca*^−/−^ mice occurs independently of ovarian hormones but requires physiological load bearing.

Endosteal responses to disuse normally change with age, such that medullary expansion with disuse occurs in mature but not growing animals (67). This may also be relevant to the age dependence of bone formation observed in the marrow of skeletally mature female *Prkca*^−/−^ mice. Disuse-induced bone loss involves suppression of Wnt signaling (65), and perturbing components of the Wnt pathway has previously been shown to have gender-specific effects on bone mass (68) and on the responses to disuse (17). The bases for these gender-specific effects are largely unknown, in part because the interactions between Wnt and androgen signaling in bone have not been studied as extensively as those between Wnt and estrogen signaling. The findings of this study suggest that androgen signaling may suppress bone phenotypes observed in female mice.

Our findings demonstrate for the first time that PKCα is a regulator of the important Wnt signaling pathway in osteoblasts. *In vivo* changes, particularly in gene expression, must be interpreted with caution, given that the model used in these studies is a germ line deletion of PKCα, although *in vitro* studies support there being cell-autonomous roles for PKCα in osteoblasts. PKCα suppresses Wnt signaling, as evidenced by the reduced proportion of β-catenin in the active form and reduced activity of the Wnt target gene alkaline phosphatase following PKC activation when PKCα is present. Because Wnt signaling is critical for osteoblast lineage commitment (69, 70), this suggests a mechanism whereby PKCα suppresses osteoblast differentiation. It is also possible that loss of PKCα may have, directly or indirectly, altered Wnt protein levels. However, given that previous publications have demonstrated that PKCα interacts with canonical Wnt signaling at the level of β-catenin in other cell types (27, 28), this possibility was not pursued in the current study.

In conclusion, deletion of *Prkca* in mice *in vivo* leads to age-related bony invasion of the medullary cavity at specific sites of the long bones in young adult female but not male mice. This invasion occurs despite ovariectomy, does not occur in the absence of functional load bearing, and is observed in orchidectomized PKCα male mice. The effects of PKCα, at least in female mice, include suppression of osteoblastic differentiation and suppression of Wnt target gene expression, revealing a novel role for PKCα in the regulation of Wnt signaling in osteoblastic cells. In the absence of PKCα, neither strain nor estradiol are capable of recruiting quiescent osteoblasts to the cell cycle, although their capacity to proliferate in response to Wnt3a is not diminished. From these data, we infer that in female, but not male, mice, PKCα acts as a suppressor of loading-related bone formation on the endosteum, with no discernible effect on the periosteum. *Prkca* deletion in female mice phenocopies some aspects of Gaucher disease in humans. As a molecular regulator of osteoblastic activity, PKCα may be a suitable target for therapeutic approaches to various bone disorders.

## Footnotes

4 The abbreviations used are:

ER: estrogen receptor
CLBOb: cortical long bone-derived mouse osteoblastic cell
PMA: phorbol 12-myristate 13-acetate
qRT-PCR: quantitative RT-PCR
μCT: microcomputed tomography
IM: intramedullary
OVX: ovariectomy
c/d: charcoal/dextran-stripped.
